# Examining health literacy disparities in the United States: a third look at the National Assessment of Adult Literacy (NAAL)

**DOI:** 10.1186/s12889-016-3621-9

**Published:** 2016-09-13

**Authors:** R. V. Rikard, Maxine S. Thompson, Julie McKinney, Alison Beauchamp

**Affiliations:** 1 Department of Media & Information, Michigan State University, 409 Comm Arts, 404 Wilson Road, East Lansing, MI 48824 USA; 2 Department of Sociology & Anthropology, North Carolina State University, Raleigh, NC USA; 3 McKinney Health Literacy Services, Cambridge, MA USA; 4 Deakin Population Health SRC, Faculty of Health, Deakin University, Melbourne, VIC Australia

## Abstract

**Background:**

In the United States, disparities in health literacy parallel disparities in health outcomes. Our research contributes to how diverse indicators of social inequalities (i.e., objective social class, relational social class, and social resources) contribute to understanding disparities in health literacy.

**Methods:**

We analyze data on respondents 18 years of age and older (*N* = 14,592) from the 2003 National Assessment of Adult Literacy (NAAL) restricted access data set. A series of weighted Ordinary Least Squares (OLS) regression models estimate the association between respondent’s demographic characteristics, socioeconomic status (SES), relational social class, social resources and an Item Response Theory (IRT) based health literacy measure.

**Results:**

Our findings are consistent with previous research on the social and SES determinants of health literacy. However, our findings reveal the importance of relational social status for understanding health literacy disparities in the United States. Objective indicators of social status are persistent and robust indicators of health literacy. Measures of relational social status such as civic engagement (i.e., voting, volunteering, and library use) are associated with higher health literacy levels net of objective resources. Social resources including speaking English and marital status are associated with higher health literacy levels.

**Conclusions:**

Relational indicators of social class are related to health literacy independent of objective social class indicators. Civic literacy (e.g., voting and volunteering) are predictors of health literacy and offer opportunities for health intervention. Our findings support the notion that health literacy is a social construct and suggest the need to develop a theoretically driven conceptual definition of health literacy that includes a civic literacy component.

## Background

Health literacy is an important determinant of health inequities across groups. The definition of health literacy is evolving from a focus on clinical risk to a focus on health literacy as an asset [1]. The clinical risk definition is prominent in the National Academy of Medicine’s (formerly the Institute of Medicine) description of health literacy as an individual’s capacity to obtain, process and understand basic health information and services needed to make appropriate health decisions [2]. The asset definition draws from public health and health promotion and directs attention to empowerment or “social and environmental factors that enable individuals to have greater control over health” [1]. More importantly, the clinical or risk approach reifies health literacy as an individual deficit instead of focusing on how social and economic factors work in tandem to impact population level health. Identifying health literacy as an asset, we explore determinants of health literacy using a relational social class model in conjunction with objective measures of social class. Objective indicators of social class signal differential access to resources but may not capture the mechanisms that link social inequalities to health literacy. In contrast, a relational view of social class asserts that connections to others through work, civic engagement, and social networks promote norms, values and benefits that facilitate obtaining and using health related information [3–5]. Relational social class represents social connections that are dynamic, fluid, and negotiable and thus are amenable to health promotion interventions. Our research contributes to knowledge of how diverse indicators of social inequalities contribute to an understanding of disparities in health literacy in the United States [6, 7]. Moreover, our research objective is consistent with an emerging body of international health literacy research examining the social gradient in health literacy [8, 9].

Research studies observe an array of sociodemographic differences in the risk of low health literacy in the United States. Low health literacy is more prevalent among the elderly, men, racial/ethnic minorities, and low socioeconomic status (SES) groups [10–13]. The overall decline in physical and mental ability associated with aging may account for lower levels of health literacy among the elderly [13–15]. However, some challenges contribute to disparities in health literacy because they are more likely among low-income elderly. These include limited activities in daily living and more rapid cognitive decline [16–18]. Some research indicates that men have lower levels of health literacy compared to women [10, 13]; however, other research suggests the opposite [19, 20]. Similarly, the connection between race/ethnicity and health outcomes consistently indicates that compared to whites, racial/ethnic minorities have higher rates of morbidity and mortality [21] and lower levels of health literacy [11, 19].

Previous research using structural or objective indicators of social class (e.g., income, education attainment, and occupational status) consistently reveals socioeconomic disparities in health literacy [10, 13, 16, 22–27]. Studies find a positive relationship between individual level of income and health literacy. For example, an increase of approximately $5000 in personal income is associated with a significant increase in health literacy levels [11, 13, 28]. Educational attainment shapes individuals’ social status by influencing accumulated knowledge, skills, resources, habits, and, in turn, health [29]. Higher levels of educational attainment translate into better overall health outcomes and higher levels of health literacy [10, 12, 20, 30, 31]. Likewise, high occupational attainment as measured by higher prestige, power or influence is associated with higher health literacy levels [32].

There are limitations to the use of income, educational attainment, and occupational status as determinants of health literacy. The rationale for using income follows that increased income provides additional access to health resources [33]. However, the relationship between income and health is confounded by reverse causality [34]; not only are poverty and low income a significant risk for poor health outcomes, but the experience of a severe chronic illness or disability may also reduce overall earning potential [17]. The connection between educational attainment and health is linear and positive. Yet, education does not completely capture access to resources, and is only significant to the extent that the associated skills and knowledge are socially valuable [4, 34]. Occupational prestige is the mutual assessment of the status associated with the title of an employment position. Occupational status, however, is only applicable for working-age adults in the labor force [3, 34] and occupational status may not capture all the social connections related to health literacy such as social empowerment. Moreover, income, educational attainment, and occupational status are of limited utility in terms of offering insight into the effect of SES position on health literacy as previous research indicates health literacy independently predicts health outcomes when controlling for structural indicators of socioeconomic position [8, 15].

Relational social class highlights the mechanisms that might reveal how individuals come to accumulate health literacy. Relational social class is the emergence of social groups as a result of interdependent economic relationships [3, 35]. Indicators of relational social class assess the relative degree of connection between individuals and larger social institutions. Class-based social relationships account for how and why members of a social class advance their economic and social well-being as well as access socially valued resources to the detriment of others [3, 36]. These indicators may include economic inclusion, political participation, and civic engagement [37, 38]. Employment status is the degree of economic inclusion that either grants or denies individuals access to needed resources. Specifically, full-time employment compared to part-time employment, unemployment, and retirement, protects and fosters perceived health and overall physical health [39, 40]. Moreover, full-time employment in the United States may include employer provided health insurance, which connects people to the system of health care. Voting is a form of political participation directly tied to the development and implementation of large scale public policy [41]. Voting may also grant people a critical sense of belonging to a community and having rights within that community [42]. Thus, voting connects people across vertical power gradients and creates ties to formal institutions [43]. Previous research reveals that individuals living in regions with high voting rates are significantly less likely to report poor self-rated health than individuals living in areas with low voter turnout [44, 45]. Civic engagement activities, such as volunteering and library use, are essential to build informal ties that may improve mental and physical health as well as access to health information [46–48]. Differential availability of and access to social resources are equally important to understand disparities in health literacy.

Social resources are normative social means that provide economic protection and benefits. Measures of social resources may include language spoken, marital status, and methods of seeking out health information [37, 38]. Speaking the primary or dominant language is an essential component to integrate and navigate within a given social context [49, 50]. In the United States, previous research reveals that Hispanics who only speak Spanish, compared to Hispanics who speak English and Spanish, experience greater disparities in use of healthcare services and health outcomes [50, 51]. Marriage or living as a married couple confers increased economic stability, lower levels of depression, and higher levels of life satisfaction compared to people who never marry [52, 53]. Access to multiple sources of health information provides the benefit of informed health decision making [10] and is associated with less ambiguous knowledge of health conditions [54].

Relational social class and social resources are examined in this study to provide greater insight into social inequality in health literacy. In general, we expect relational and social resource measures will have additional value in understanding the disparities in health literacy. Thus, we hypothesize that relational social class measures (i.e., full-time employment, voting, and volunteering) are significant positive predictors of higher health literacy levels, net of the demographic and objective SES (i.e., income, educational attainment, and occupational prestige) resources measures. We also hypothesize that access to social resources (i.e., native English speaker, marriage, and the number of health information sources) is significantly associated with higher levels of health literacy, net of the demographic and objective SES resources measures.

## Method

### Data source

Data come from the 2003 National Assessment of Adult Literacy (NAAL). The NAAL is a unique nationally representative data set that includes an Item Response Theory (IRT) based measure of health literacy as well as measures of relational social class, social resources, and objective social resources. The written informed consent for participation in the NAAL, sampling design, and complete sample size are described elsewhere [55]. The complete NAAL household sample of more than 18,000 respondents was limited to adults (i.e., 18 years of age and older) for an analytical sample of 14,592 respondents. The analyses were performed with approval from the Institutional Review Board at North Carolina State University.

### Measures

#### Dependent variable

The health literacy measure is based on Item Response Theory which models the level of difficulty for a given item and an individual’s performance (i.e., answering the question correctly) on the item [56]. Baldi & colleagues [57] developed 28 of the 152 items to measure health literacy in the NAAL. Responses to 23 of the 28 items were originally scored correct, incorrect, omitted, or not reached (i.e., 1 = correct, 0 = incorrect, 8 = omitted, and 9 = not reached) and responses to 5 items were scored correct, partially correct, incorrect, omitted, or not reached (i.e., 1 = correct, 2 = partially correct, 0 = incorrect, 8 = omitted, and 9 = not reached). We recoded omitted and not reached responses as incorrect and employed the more general Graded Response Model (GRM) [58] IRT model to estimate health literacy scores [59]. The IRT parameters and recoded item responses were entered into VisualDF [60]^1^ to compute the health literacy values.

### Independent variables

#### Demographic

Respondents’ age was measured in years and was computed from information on the respondent’s date of birth and the date of the NAAL assessment. Respondent’s sex was coded as a dummy variable (i.e., 0 = Male, 1 = Female) with males as the excluded category. Responses to three questions determine respondents’ race/ethnicity. The first question asks: “Are you Hispanic or Latino?” The second question asks respondents their country of birth. Hispanic/Latino respondents born in the United States are coded as native born while respondents’ born outside the United States are foreign born. If the respondent indicated that they were not Hispanic/Latino, then the third question asked respondents’ to indicate their racial/ethnic group [61]. Each race/ethnic group category was coded as a separate dummy variable and white respondents were the excluded category in the analyses.

### Socioeconomic resources

A single question item asked respondent’s approximate total household income for the past 12 months. The original 15 household income categories were collapsed into eight categories, recoded to the median value of each category, and rescaled in thousands of dollars [62]. Recoding and rescaling household income provides an intuitive interpretation for the effect of median household income in thousands of dollars rather than in units of the categories [63]. To determine income from savings and investments, two questions asked if the respondent or anyone in the respondent’s household received: 1) interest from savings or other bank accounts (other than dividends) and 2) dividend income from stocks or mutual funds or income from rental property, royalty, estates, or trusts. Response options for both questions are: 1) Yes, me; 2) Yes, someone else; 3) Yes, someone else and me; and 4) No. Affirmative responses to the two questions are recoded into dummy variables and summed. A derived measure of educational attainment includes 11 response options that served as a measure of educational attainment. Occupational status was recoded into corresponding 2000 Census Occupational Codes by NCES staff. In the analyses the Census Occupational Codes were recoded into Nam-Powers-Boyd Occupational Status Scale values that range from 1 (lowest status) to 100 (highest status) [64–67].

### Relational social class

Employment status is a derived variable and the original six response categories include: 1) Employed Full Time, 2) Employed Part Time, 3) Employed Not at Work, 4) Unemployed Looking for Work, 5) Unemployed Not Looking for Work, and 6) Out of Labor Force. The first three response options were collapsed into an “Employed” category. The last three response options were collapsed into an “Unemployed” category. In our analyses, “Unemployed” was the excluded category. A derived measure of voting in the 2000 election included the response options: 1) Not a Citizen 2) Did Not Vote, 3) Voted, and 4) Do Not Remember. Response categories are recoded into dummy variables and respondents who voted were the excluded category in the analyses.

Respondents indicated the amount of information about current events, public affairs and the government from six sources including: 1) Newspapers, 2) Magazines, 3) Internet, 4) Radio and Television, 5) Books or Brochures, and 6) Family Members, Friends, or Co-workers. The response options for each source of information were: 1 = A Lot, 2 = Some, 3 = A Little, and 4 = None. Response options were reverse coded and higher values indicate a greater amount of information and then averaged across the six sources for non-missing values only. Respondents were asked how often they used a library for any reason. Response options included: 1) Daily, 2) Weekly, 3) Monthly, 4) Once or Twice a Year, and 5) Never. To create equivalent library use categories, the first three responses were collapsed and recoded into a dummy variable for “Frequent” library use. The response options Once or Twice a Year and Never were each recoded into dummy variables. In the analyses, “Never” was the excluded category.

Two question items provide the measure of volunteer activity. First, respondents were asked if they gave any unpaid time as a volunteer to a group or organization during the past year. The response options were recoded into a dummy variable (i.e., 0 = No, 1 = Yes). Second, if the respondent volunteered a follow up question asked how often they volunteered and included the response options: 1) Most days, 2) A few days a week, 3) About once a week, and 4) Less than once a week. The combined response options were reverse coded so higher values indicate a higher level of volunteer activity. To create equivalent volunteer activity categories, the response options Most days, A few days a week, and About once a week were collapsed and recoded into a dummy variable for “Frequent” volunteer activity. The response options Less than once a week and Never were each recoded into dummy variables. In the analyses, the “Never” volunteer was the excluded category.

### Social resources

Language spoken is a derived measure from responses to two questions: “When you were growing up, what language or languages were usually spoken in your home?” and “Which language do you often speak now?” Respondents who indicated English for both questions were recoded as only English speaking. Respondents who spoke Spanish growing up and now speak English were recoded as Spanish/English as Second Language (ESL). Respondents who spoke Spanish growing up and only spoke Spanish were recoded as Speaking Spanish Only. The remaining respondents who spoke any other language growing up and now speak English were recoded as Other Language Spoken ESL. Native English speaking respondents are the excluded category in the analyses.

Marital status is a derived measure and respondents were categorized as either: 1) Never Married, 2) Married or Living as Married, and 3) Separated/Divorced/Widowed. The three categories were recoded into dummy variables and married or living as married was the excluded category. Respondents indicated the amount of information about health issues obtained from six sources. The sources included: 1) Newspapers, 2) Magazines, 3) Internet, 4) Radio and Television, 5) Books or Brochures, and 6) Family Members, Friends, or Co-workers. The response options for each source of information were: 1 = A lot, 2 = Some, 3 = A little, or 4 = None. Cronbach’s alpha analysis revealed Newspapers, Magazines, and Books or Brochures are a reliable measure for source of health information (α = 0.71). Response options were reverse coded and higher values indicate a greater amount of information and then averaged for the three sources for non-missing values only.

### Data analysis

Two analytical techniques were employed in the research. First, weighted descriptive statistics were estimated for all variables. Bivariate correlation matrices (available upon request) do not reveal multicollinearity among the predictor variables. Second, a series of weighted multivariate Ordinary Least Squares (OLS) regression analyses were used as the dependent variable was a continuous measure. Given the multi-stratified cluster sampling design in the NAAL, all analyses were conducted in STATA 12 [68] to include sampling weights.

## Results

### Descriptive statistics

Table 1 displays the weighted descriptive statistics for all variables. On average, respondents were 45 years of age, female, and white. The average median household income was approximately $49,000 and more than half of respondents received additional income from savings and/or investments. The majority of respondents were high school graduates, employed, and voted in the 2000 election. On average, respondents obtained a lot of their information about current affairs from multiple sources and approximately a third of respondents frequently used a library but the majority did not volunteer. On the whole, respondents were native English speakers, married or living as a married couple, and obtained a lot of their health information from multiple sources.

**Table 1:** Descriptive statistics for demographic, socioeconomic resources, and health literacy score (Weighted)^a^

Demographic	Mean	Lin. Std. Err.	[95 % Conf. Interval]		Min	Max
Age	44.9	0.29	44.33	45.48	18.00	100.00
Men	0.48	0.01	0.46	0.49	0.00	1.00
Women	0.52	0.01	0.51	0.54	0.00	1.00
White	0.71	0.02	0.67	0.74	0.00	1.00
African-American	0.12	0.01	0.09	0.14	0.00	1.00
Foreign Born Hispanic/Latino	0.08	0.01	0.06	0.10	0.00	1.00
Native Born Hispanic/Latino	0.05	0.01	0.03	0.06	0.00	1.00
Asian/Pacific Islander	0.03	0.01	0.02	0.04	0.00	1.00
American Indian/Alaskan Native	0.01	0.00	0.00	0.01	0.00	1.00
Multiracial	0.01	0.00	0.01	0.02	0.00	1.00
Socioeconomic Resources						
Median Household Income (in $1000)	48819.66	1001.99	46840.24	50799.09	0.00	124999.50
Num. Inc. Sources From Savings/Investments	0.69	0.02	0.65	0.74	0.00	2.00
Educational Attainment	5.59	0.08	5.44	5.74	1.00	11.00
Nam Powers Boyd Occupational Score	53.36	0.56	52.25	54.46	0.00	100.00
Relational Social Class						
Employment Status						
Employed	0.66	0.01	0.64	0.67	0.00	1.00
Unemployed/Out of Labor Force	0.34	0.01	0.33	0.36	0.00	1.00
Voted in 2000						
Not a Citizen	0.08	0.01	0.06	0.10	0.00	1.00
Did Not Vote	0.30	0.01	0.29	0.32	0.00	1.00
Voted	0.58	0.01	0.56	0.60	0.00	1.00
Do Not Remember	0.04	0.00	0.03	0.04	0.00	1.00
Current Affairs/Political/Govt. Info.	2.64	0.01	2.62	2.66	1.00	4.00
Source						
Library Use						
Frequent	0.33	0.01	0.32	0.35	0.00	1.00
Occasional	0.33	0.01	0.31	0.34	0.00	1.00
Never Use Library	0.34	0.01	0.32	0.36	0.00	1.00
Volunteer Activity						
Frequent	0.17	0.01	0.16	0.18	0.00	1.00
Occasional	0.21	0.01	0.19	0.22	0.00	1.00
Never	0.62	0.01	0.60	0.64	0.00	1.00
Social Resources						
Language Spoken						
Native English Speaker	0.84	0.01	0.81	0.87	0.00	1.00
Spanish/ESL	0.04	0.00	0.03	0.04	0.00	1.00
Speaks Only Spanish	0.05	0.00	0.04	0.06	0.00	1.00
Other Language Spoken ESL	0.08	0.01	0.06	0.10	0.00	1.00
Marital Status						
Never Married	0.21	0.01	0.19	0.22	0.00	1.00
Married/Living as Married	0.60	0.01	0.59	0.62	0.00	1.00
Separated/Divorced/widowed	0.19	0.01	0.18	0.20	0.00	1.00
Health Information Source	2.49	0.01	2.46	2.51	1.00	4.00
Health Literacy Score	243.10	0.86	241.39	244.81	89.74	336.38

^a^ *Source*: The National Assessment of Adult Literacy (NAAL) 2003. Respondents 18 years of age and older. *N* 14,592

### Multivariate analyses

A series of weighted Ordinary Least Squares (OLS) regression models predicting health literacy level are displayed in Table 2. Examining the coefficient for measures in Model 1, age was significant and negatively associated with health literacy level. Moreover, age maintains a negative, significant, and robust association with health literacy across Models 1–5. Women’s health literacy scores were nearly five points higher than the scores of men (Model 1). The pattern remained consistent across Models 1–5. African Americans, Foreign Born Hispanic/Latinos, and Native Born Hispanic/Latinos have health literacy scores that were significantly lower compared to Whites (Model 1). Next, the coefficients for Asian/Pacific Islanders, Native Americans, and Multiracial respondents were also lower than the scores for Whites, but this pattern was explained by variables in the full model (Model 5), in particular the social resource measures. In the full model, the baseline coefficient for Asian/Pacific Islanders was reduced by 58 and 54 % for Native Americans. The overall model accounts for 13.6 % of the variation in the health literacy scores.

**Table 2:** Ordinary least squares regression of health literacy score on demographic, socioeconomic resources, & social resources (Weighted)^a^

	Model 1	Model 2	Model 3	Model 4	Model 5
Demographic					
Age	−0.567***	−0.569***	−0.472***	−0.493***	−0.518***
	(−0.230)	(−0.230)	(−0.191)	(−0.200)	(−0.210)
Female	4.713***	6.583***	5.968***	5.281***	4.924***
	(0.056)	(0.079)	(0.071)	(0.063)	(0.059)
African American	−25.90***	−17.02***	−16.31***	−16.52***	−16.48***
	(−0.195)	(−0.128)	(−0.123)	(−0.124)	(−0.124)
Foreign Born Hispanic/Latino	−44.49***	−33.59***	−27.96***	−22.22***	−11.45***
	(−0.279)	(−0.211)	(−0.176)	(−0.139)	(−0.072)
Native Born Hispanic/Latino	−18.36***	−10.85***	−9.265***	−9.534***	−5.620*
	(−0.092)	(−0.054)	(−0.046)	(−0.048)	(−0.028)
Asian/Pacific Islander	−8.776*	−8.474*	−12.78***	−9.313**	−3.665
	(−0.037)	(−0.036)	(−0.054)	(−0.039)	(−0.015)
American Indian/Alaskan Native	−22.58**	−12.00	−11.36	−11.76	−10.43
	(−0.045)	(−0.024)	(−0.023)	(−0.024)	(−0.021)
Multiracial	−13.04**	−8.315*	−7.476*	−7.571*	−7.265+
	(−0.037)	(−0.024)	(−0.021)	(−0.022)	(−0.021)
Socioeconomic Resources					
Median Household Income (in $1000)		0.248***	0.131***	0.116***	0.108***
		(0.219)	(0.116)	(0.103)	(0.095)
Income From Savings/Investments		6.391***	3.517***	3.011***	3.053***
		(0.125)	(0.069)	(0.059)	(0.060)
Educational Attainment			3.810***	3.318***	3.281***
			(0.264)	(0.230)	(0.227)
Nam Powers Boyd Occupational Score			0.0663***	0.0481*	0.0465*
			(0.055)	(0.040)	(0.039)
Relational Social Class					
Employed				1.757	1.437
				(0.020)	(0.016)
Voted in 2000					
Not a Citizen				−9.106***	−2.797
				(−0.059)	(−0.018)
Did Not Vote				−3.925**	−3.567**
				(−0.043)	(−0.039)
Do Not Remember				−1.728	−1.231
				(−0.008)	(−0.005)
Current Aff/Pol/Govt. Info				1.523+	0.704
				(0.019)	(0.009)
Library Use					
Frequent Library Use				4.118**	4.098**
				(0.046)	(0.046)
Occasional Library Use				5.983***	5.642***
				(0.067)	(0.063)
Volunteer Activity					
Frequently Volunteer				3.412*	3.200+
				(0.031)	(0.029)
Occasionally Volunteer				2.238	1.820
				(0.022)	(0.018)
Social Resources					
Language Spoken					
Spanish/ESL					−5.824+
					(−0.025)
Speaks Only Spanish					−9.885*
					(−0.051)
Other Language Spoken ESL					−19.11***
					(−0.121)
Marital Status					
Never Married					−3.574**
					(−0.034)
Separated/Divorced/Widowed					−0.780
					(−0.007)
Health Information Source					0.929
					(0.017)
Constant	274.6***	253.9***	232.2***	230.3***	233.8***
R-squared	0.136	0.213	0.270	0.279	0.286
F	101.4***	231.9***	252.2***	160.3***	126.1***

^a^ *Source*: The National Assessment of Adult Literacy (NAAL) 2003. Unstandardized coefficients above standardized coefficients in parentheses. Respondent *N* = 14,592. + *p* < 0.05 * *p* < 0.01 ** *p* < 0.001 *** *p* < 0.0001

Health literacy level increased by a quarter of a point for every additional thousand dollars ($1000) in median household income and increased six points with each additional source of income from savings and investments (Model 2). The addition of income and wealth measures accounted for 21.3 % of the variation in health literacy scores which represents a 56.6 % increase over the baseline model. Respondents’ health literacy scores increased by nearly four points for each additional level of educational attainment, net of other variables in the model (Model 3). As an individual’s occupational prestige increased there was approximately a one-point increase in health literacy scores. The addition of educational attainment and occupational prestige reduced the effect of income from savings and investments by 44.91 %; but the effect remains statistically significant. Overall, Model 3 accounted for 27.0 % of the variation in health literacy scores.

The measures of civic engagement voting behavior, library use, and volunteering are significant predictors of health literacy (Model 4). Lower health literacy scores were observed for people who were either not citizens or did not vote. The significant negative association remained consistent for those who did not vote (Model 5). Frequent and occasional library use was associated with a four and five point increase, respectively, in health literacy scores (Models 4 and 5). Health literacy scores increased by three points for respondents who frequently volunteered (Model 4) and the significant positive association remained consistent in Model 5.

Health literacy scores were significantly lower for those who grew up speaking Spanish and now speak English, those who speak Spanish only, and those who grew up speaking a language other than English or Spanish (Model 5). Health literacy was significantly lower for respondents who never married compared to married or co-habitating respondents. Model 5 accounted for 28.6 % of the variation in health literacy scores which represented a 110.3 % increase over the baseline model. Overall, the results suggest that measures of relational social class and social resources improve our understanding of disparities in health literacy.

## Discussion

In this study, we identify social determinants of low health literacy as well as individual assets that promote health literacy. In particular, our findings add new insights on the connection between relational social class and health literacy. Voting, volunteering, and library use are associated with higher health literacy levels, net of demographic and objective SES resources measures. Zarcadoolas and colleagues [69] multidimensional model of health literacy emphasizes the importance of political or civic literacy. Civic literacy enables citizens to become aware of health issues and involved in the decision-making processes through civic and social channels. Our results suggest that voters may act to support or oppose governmental influence on health policy. The positive effect of civic engagement on health literacy suggests that participation in important civic activities like voting and volunteering involve connecting with one’s community in ways that promote health literacy or attention to health affairs. Civic engagement creates social ties by connecting individuals to a larger community or movement. In turn, social connections provide an opportunity to become aware of as well as increase confidence and willingness to seek out available health resources. Understanding how positive health literacy flows from civic engagement may offer a counterpoint to a risk approach to health literacy which focuses on a deficit model.

The findings also reveal that social resources are associated with higher health literacy. The findings related to language are mostly what we would expect; yet, there are some finer points worth noting. The majority of health and healthcare information in the United States is written and spoken in English and translations of the information are also widely available in Spanish. Therefore, as a social resource, it is not surprising that respondents who learn English in childhood have a relative advantage over those who are learning or do not understand English. What is surprising is that Spanish speakers who have *not* learned English have significantly higher skills than those from other language groups who *have* learned English. The finding suggests two points of interest. First, providing information in Spanish is important for the Spanish speaking community. Second, the language instruction currently in place for speakers of other languages may not do enough to bring them up to the health literacy levels of others. The results support continued and improved efforts in creating multilingual health information and providing free and high quality ESOL (English for Speakers of Other Languages) in adult education programs [70].

Marriage is a social resource that benefits health literacy by encouraging healthy behaviors. The gender and health literature provides insight into the relationship between marital status and health literacy. Marriage, or living as a married couple, fosters social interaction and an expansion of social networks as well as economic support to pay for healthcare services [52, 53]. It is also possible that, because women maintain more consistent social relationships throughout life, social connections provide women with the same types of benefits that we see for people who are married or living as married. Social interaction tends to improve one’s health literacy skills and subsequently prolong one’s lifespan.

Consistent with previous research, we found that as people age their health literacy declines, that health literacy is higher for women compared to men, and that racial and ethnic minorities have lower health literacy compared to whites. The underlying assumption is that the lower health literacy for ethnic minorities is due to lower socioeconomic status, lower educational attainment, employment status, and language barriers. However, our results show that, minorities have consistently lower health literacy scores when compared to whites who with the same educational attainment, income, gender and age. The disadvantage of being a minority cuts across many of the protective features that might account for their lower health literacy. Other studies have not revealed this persistence in health literacy disparities attributable solely to racial/ethnic disparities. Our findings reveal that nativity has a protective influence, even for the most disadvantaged racial groups. While both native born and foreign born Hispanics had lower health literacy levels, foreign born Hispanics had significantly lower scores than even their native born counterparts. The differences may be a result of native born Hispanics’ assimilation of language and culture in the United States.

Household income and additional income from savings and/or investments are each independently associated with a significant increase in health literacy scores. What is notable, however, is that the association with higher scores is greater for these additional sources of wealth than it is for an overall increase in median household income. Health literacy scores increased by a quarter of a percent for every additional $1000 in median household income; yet, each additional source of wealth from savings and/or investments is associated with a six full point increase in health literacy scores. The significant and positive effect of increasing levels of educational attainment concurs with previous research that education has an independent and positive relationship with individual levels of health literacy [10, 12, 20, 30].

The limitations of the existing research should be considered when interpreting findings. First, the NAAL health literacy question items only focus on the functional aspect of health literacy and do not provide an assessment of oral communication ability and/or critical analytic ability. In addition, the assessment does not reflect the ability to communicate or the ability to determine the quality of health information and how an individual might make decisions or change behavior based on health information. Second, the NAAL is a secondary cross-sectional data set collected over a decade ago. Thus, the present analyses cannot address causal relationships; instead, a longitudinal research design is appropriate to examine how change in social, economic, and structural factors impact change in health literacy level over time [71, 72]. Moreover, we caution that our results may not reflect the relationship between health literacy disparities and health inequities in the United States given the systemic changes in the system of health care as a result of provisions in the 2010 Patient Protection and Affordable Care Act [73, 74]. Despite these limitations, the present research adds significant value to clarify the distribution of disparities in health literacy and identify opportunities to reduce inequity.

## Conclusions

Health literacy may be viewed as several integrated concepts relating to what people need to be able to participate in effective decision-making about health for themselves, and their families [75]. Earlier definitions, such as the National Academy of Medicine’s [2], focus on individual deficits (and subsequent risk) within clinical settings [76]. Definitions from other countries conceptualize health literacy as an asset for good health, not just in clinical settings, but in everyday life [31, 77]. Our findings support a broader understanding of health literacy in the United States.

The results presented herein provide strong evidence that disparities in health literacy cut across demographic and socioeconomic groups, level of civic engagement through voting and volunteering, and available social resources. To increase equity in health literacy and health outcomes will necessitate a fundamental shift in perspective among health literacy practitioners and researchers in the United Sates. There remains a critical need to develop a theoretically driven conceptual definition of health literacy as a social construct [78–80]. Instead of focusing on health literacy as an individual deficit; a shifting perspective of health literacy emphasizes the importance of social context, the role of social interaction, and the creation of social connections as an asset. These new insights in disparities in health literacy can provide a focus for creating relevant interventions. For example, interventions to increase health literacy may include components to improve self-efficacy, personal empowerment, civic engagement and social interactions. Health literacy interventions must focus on how health literacy skills are used on a daily basis. There is a need for continuing efforts to improve dissemination of health information in plain language, Spanish and other languages, and improve the quality and accessibility of English language and literacy instruction.

## Abbreviations

2PL: Two parameter logistic
CTT: Classical test theory
GRM: Graded response model
IRT: Item response theory
NAAL: National assessment of adult literacy
NCES: National center for education statistics
OLS: Ordinary least squares.

## Footnotes

1 VisualDF is a free, open-source program available at http://www4.ncsu.edu/~awmeade/VisualDF/Main.htm to compute effect size indices of differential functioning of items and test scales.
